# Optimizing delivery of HIV-1 conserved region-derived immunogen for induction of T and B cell responses in rhesus macaques

**DOI:** 10.1186/1742-4690-9-S2-P31

**Published:** 2012-09-13

**Authors:** M Rosario, G Koopman, A Mbewe-Mvula, ML Knudsen, ED Quakkelaar, N Borthwick, R Wagner, DA Price, P Liljestrom, CJ Melief, JW Drijfhout, S Colloca, A Nicosia, T Hanke

**Affiliations:** 1 University of Oxford, Oxford, UK

## Background

The complexity of candidate HIV-1 vaccine formulations is increasing due to extreme challenges faced when trying to prevent or control HIV-1 infection.

## Methods

Immunogen HIVconsv based on the most conserved regions of the HIV-1 proteome was used to explore combinations of seven distinct vaccines modalities in heterologous prime-boost regimens delivered to rhesus macaques to optimize induction of T cell and antibody responses. These include plasmid DNA (P), Semliky Forest virus replicons delivered as DNA (DREP; D) or virus particles (VREP; V), modified vaccinia virus Ankara (MVA; M), adenoviruses of human (HAdV-5; A) and chimpanzee origin (ChAdV-63; C) and adjuvanted synthetic long peptides (SLP; S).

## Results

A number of observations were made. Thus, a very potent combination for induction of HIV-1-specific T cells was an adenovirus vector (A or C) followed by poxvirus M. S boost broadened T cell responses, but did not prime T cells efficiently. D was a stronger prime than P. PPP was the best prime for T cells, while PSS was best for induction of antibodies. Even very complex regimen PPPAMSSCMV continued to recruit new T cell clones into the response to a single epitope, although a ceiling for immunodominant responses was reached; subdominant responses could be boosted up to the last V delivery. Finally, PPSS, but not SSSS could protect 2/6 animals from SIVmac251 acquisition.

## Conclusion

These results will guide initial design of human trials. So far, human studies in Oxford testing CM, PPPCM and PPPMC regimen concur with observations made in rhesus macaques.

